# Ginsenoside Rh2 Ameliorates Lipopolysaccharide-Induced Acute Lung Injury by Regulating the TLR4/PI3K/Akt/mTOR, Raf-1/MEK/ERK, and Keap1/Nrf2/HO-1 Signaling Pathways in Mice

**DOI:** 10.3390/nu10091208

**Published:** 2018-09-01

**Authors:** Yung-Hung Hsieh, Jeng-Shyan Deng, Yuan-Shiun Chang, Guan-Jhong Huang

**Affiliations:** 1 Department of Chinese Pharmaceutical Sciences and Chinese Medicine Resources, China Medical University, Taichung 413, Taiwan; hyh3033@gmail.com (Y.-H.H.); yschang@mail.cmu.edu.tw (Y.-S.C.); 2 Department of Pharmacy, Kuang Tien General Hospital, Taichung 433, Taiwan; 3 Taichung City New Pharmacist Association, Taichung 420, Taiwan; 4 Department of Food Nutrition and Health Biotechnology, Asia University, Taichung 413, Taiwan; dengjs@asia.edu.tw

**Keywords:** ginsenoside Rh2, lipopolysaccharide, acute lung injury, MEK, Nrf-2

## Abstract

The anti-inflammatory effect of ginsenoside Rh2 (GRh2) has labeled it as one of the most important ginsenosides. The purpose of this study was to identify the anti-inflammatory and antioxidant effects of GRh2 using a lipopolysaccharide (LPS) challenge lung-injury animal model. GRh2 reduced LPS-induced proinflammatory mediator nitric oxide (NO), tumor necrosis factor-alpha, interleukin (IL)-1β, and anti-inflammatory cytokines (IL-4, IL-6, and IL-10) production in lung tissues. GRh2 treatment decreased the histological alterations in the lung tissues and bronchoalveolar lavage fluid (BALF) protein content; total cell number also reduced in LPS-induced lung injury in mice. Moreover, GRh2 blocked iNOS, COX-2, the phosphorylation of IκB-α, ERK, JNK, p38, Raf-1, and MEK protein expression, which corresponds with the growth of HO-1, Nrf-2, catalase, SOD, and GPx expression in LPS-induced lung injury. An in vivo experimental study suggested that GRh2 has anti-inflammatory effects, and has potential therapeutic efficacy in major anterior segment lung diseases.

## 1. Introduction

Acute lung injury (ALI) has a high mortality rate due to shock, sepsis, ischemia reperfusion, aspiration of gastric contents, major trauma, and acute pancreatitis [1]. ALI induced either by hypoxic and ischemic stresses or bacterial endotoxins, such as lipopolysaccharide (LPS), which occurs with stable redox states, can cause DNA damage, and oxidative protein and lipid effects [2]. The main features of ALI include the leakage of plasma proteins into alveolar space, accumulation of inflamed cells, pulmonary interstitial edema, and destruction of epithelial-barrier integrity [3,4]. The activation of macrophages initiates the inflammatory response in LPS-induced ALI by releasing proinflammatory cytokines (tumor necrosis factor-alpha (TNF-α)), interleukin ((IL)-1β and IL-6), anti-inflammatory cytokines (IL-4 and IL-10), and proinflammatory mediator (NO) that are related to the tissue-injury sites in immune cell recruitment [5]. Toll-like receptor 4 (TLR4) is a transmembrane protein that is part of the pattern-recognition receptor for LPS from Gram-positive bacteria. TLR4 activates the mitogen-activated protein kinase (MAPK) and nuclear factor κB (NF-κB) to induce the production of inflammatory mediators [6]. TLR4 is a potential therapeutic marker that could be used to reduce inflammatory responses in ALI. Moreover, the phosphatidylinositol-3 kinase (PI3K)- protein kinase B (Akt)-mechanistic target of rapamycin (mTOR) signaling pathway is an important intracellular mediator, critical for the regulation of cell survival and proliferation [6,7]. The PI3K/Akt/mTOR signal pathway has been implicated in the inflammatory disease, and its inhibition is an aspect of beneficial therapy. In addition, oxidative stress signaling is involved in modulating the LPS-induced inflammatory reaction through transcription factors NF-E2-related factor 2 (Nrf2) activation. Heme oxygenase-1 (HO-1) induction by Nrf2 protects against the cytotoxicity of various oxidative stresses and inflammatory response. HO-1 is a stress-regulated protein produced through the Keap1/Nrf2/HO-1 pathway [7]. The Raf/MAPK kinase (MEK)/ERK signaling cascade has been previously characterized in response to cell growth, proliferation, and survival [8]. The Raf/MEK/ERK signal pathway is detected after LPS-induced ALI; however, the effects of modulating the pathway in vivo are unknown.

The main active constituents of ginseng (the root of *Panax ginseng* Meyer; Korean ginseng) are ginsenosides that are receiving considerable attention in the field of traditional medicine due to their potential beneficial properties on human health, including anti-inflammatory, antioxidant, antidiabetic, antitumor, immunological regulation, and slowing the aging process [9,10] properties. 

Ginsenoside Rh2 (GRh2), having a dammarane skeleton, is a rare ginsenoside. GRh2 is only found in red ginseng and has anti-inflammatory and antitumor effects, and improves memory and liver function [11]. GRh2 has antitumor activity against leukemia, prostate cancer, pancreatic cancer, and glioblastoma [12]. GRh2 has also reduced allergic reactions, and improved atopic and contact dermatitis by inhibiting the NF-κB activation, p38 MAPK phosphorylation, and inflammatory cytokines [13,14].

Several signal transduction pathways have been suggested to explain the activation of inflammatory mediators after LPS-induced ALI. Therefore, we hypothesized that the TLR4/ PI3K/Akt/mTOR, Keap1/Nrf2/HO-1, and Raf-1/MEK/ERK signaling pathway is involved the inflammatory mechanisms of GRh2 after LPS-induced ALI. Thus, the purpose of this study was to determine the anti-inflammatory and antioxidant effects of GRh2. Our results suggest that GRh2 has potential as a dietary supplement for preventing acute lung injury and inhibiting inflammation. 

## 2. Materials and Methods

### 2.1. Reagents

**Figure 1:**
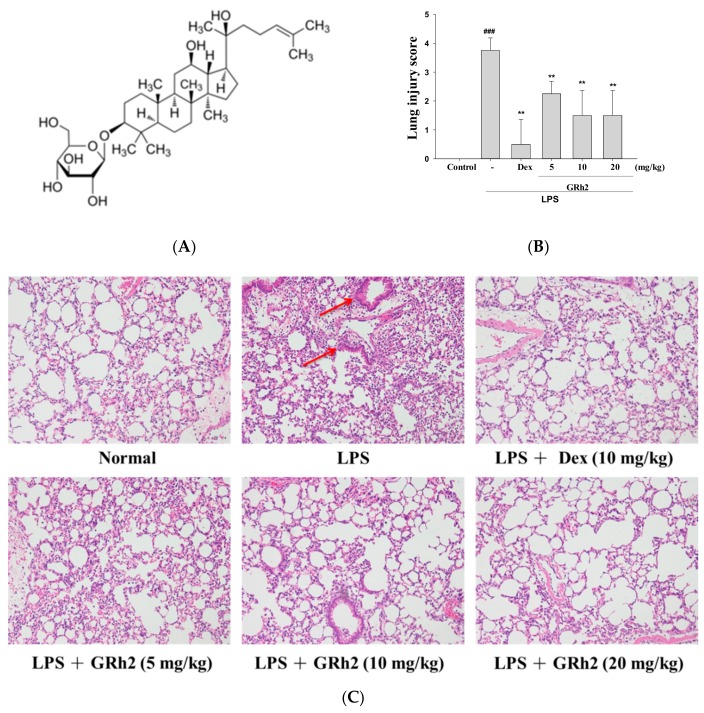
(**A**) The chemical structure of ginsenoside Rh2 (GRh2); (**B**) the lung-injury scores were determined; and (**C**) the effects of GRh2 (5, 10, and 20 mg/mL) on lipopolysaccharide (LPS)-induced lung histopathologic changes in mice. At 6 h after LPS challenge, lungs in each group were prepared for histological evaluation. Representative histological section of the lungs was stained by hematoxylin and eosin (H and E) staining, magnification (400×). The data are presented as the means ± S.E.M (n = 5). ### denotes *p* < 0.001 compared with sample of control group. ** *p* < 0.01 compared with the LPS-alone group. The red arrow indicates the symptoms of bleeding and inflammatory cell infiltration. Dexamethasone (Dex).

GRh2 (purity >98%) was supplied by Professor Yuan-Shiun Chang (Figure 1A). LPS (endotoxin from *Escherichia coli*, serotype 0127:B8), dexamethasone (Dex), and other reagents and solvents were acquired from Sigma-Aldrich (St. Louis, MO, USA). Assay kits for the determination of mouse TNF-α (430905), IL-1β (432605), IL-4 (43105), IL-6 (431305), and IL-10 (431415) (ELISA Max^TM^ Set Deluxe Kits) were received from BioLegend Inc. (San Diego, CA, USA). Primary antibodies against COX-2 (GTX60935), p-JNK (GTX52328), catalase (GTX110704), GPx (GTX116040), SOD (GTX100554), Trx (GTX100554), KAP1 (GTX102226), Keap1 (GTX60660) and TLR4 (GTX113024), AKT (GTX121937), Raf (GTX107763), and Mek (GTX121942) were purchased from GeneTex (San Antonio, TX, USA). Antibodies against PI3k (06-195) and p-AKT (04-736) were purchased from Merck Millipore (Merck KGaA, Darmstadt, Germany). Antibodies against iNOS (ab15323), NF-κB (ab16502), IκBα (ab32518), p38 (ab31828), HO-1 (ab13243), Nrf-2 (ab62352), and β-actin (ab8227) were purchased from Abcam (Cambridge, UK, USA). Antibodies against JNK (9252), p-ERK (9101), ERK (9102), p-p38 (9211), p-IκB-α (9246), mTOR (2983), p-mTOR (5536), p-Raf (9421), and p-Mek (9121) were purchased from Cell Signaling Technology (Beverly, MA, USA). Protein assay kit (5000006) (Bio-Rad Laboratories Ltd., Watford, Herts, UK) was obtained as indicated. Poly-(vinylidene fluoride) membrane (PVDF) (IPVH00010) (Immobilon-P) was obtained from Millipore Corp. (Bedford, MA, USA).

### 2.2. Animals

Male Institute of Cancer Research (ICR) mice, weighing 20–25 g, were purchased from BioLASCO Taiwan Co., Ltd. (Taipei, Taiwan). All animal procedures were conducted in accordance with the animal-management committee of the China Medical University (IACUC approval number: 104-93-N). Every effort was made to minimize suffering of the animals and reduce the number of animals used.

### 2.3. Experimental Design

Mice were randomly selected and divided into the following six groups: control, LPS only, GRh2 (5, 10, and 20 mg/kg) + LPS, and Dex (10 mg/kg; a positive drug) + LPS treatment groups (*n* = 5 in each group). ALI was induced by intratracheal instillation of LPS (5 mg/kg; 50 μL in sterile saline), then GRh2 and Dex were injected intraperitoneally 1 h prior to LPS administration. The animals were sacrificed 6 h later and the sample was collected [6].

### 2.4. Bronchoalveolar Lavage Fluid (BALF) Collection and Cell Count

BALF was collected from each individual mouse by lavaging the lung with normal saline three times and supernatants were then collected for later analysis by ELISA and protein study. The pellets were resuspended in saline for total cell counts using a hemocytometer. The sediment was resuspended to determine the total number of cells and protein content. Total cell number was determined using a hemocytometer.

### 2.5. Nitrites Assay

Determination of the nitrite level in BALF was performed using Griess reagent [7]. Briefly, we added equal volumes of Griess reagent and BALF solution (1:1) and mixed the solution. After 10 min of incubation, the absorbance of supernatants was measured by a microplate photometer plate reader at 540 nm. 

### 2.6. Histopathological Analysis

The lobe of the right lung was excised for histopathological analysis. The lung slices were fixed in 4% paraformaldehyde and dehydrated from water through a standard-graded alcohol and embedding in paraffin. Histologic specimens of lung tissue were stained with hematoxylin and eosin (H and E) stain and observed with a light microscope. The severity of lung-injury scores, from 0 to 5, depended on the degree of inflammatory infiltration, neutrophils, and dissemination, which ranged from 0 to 5. A score of 0 expressed normality; 1 expressed minimal (<1%); 2 expressed slight (1%–25%); 3 expressed moderate (26%–50%); 4 expressed moderate/severe (51%–75%); and 5 expressed severe/high (76%–100%) lung injury [4].

### 2.7. Cytokine Assay

Inflammatory profiles (TNF-α, IL-1β, IL-4, IL-6 and IL-10) of BALF were evaluated by an enzyme-linked immunosorbent assay (ELISA) system (BioLegend, San Diego, CA, USA) according to the manufacturer’s instructions. The samples were centrifuged (3000 g for 10 min at 4 °C), then stored at –80 °C for ELISA testing. In brief, to assess the level of TNF-α, IL-1β, IL-4, IL-6, and IL-10 in the BALF, 96-well plates were coated with capture antibody in ELISA coating buffer and incubated overnight at 4 °C. The plates were then washed with phosphate-buffered saline (PBS) with 0.05% Tween 20 (PBS-T) and blocked with 10% FBS in PBS for 1 h at room temperature. Serial dilutions of standard antigen or sample in dilution buffer (10% FBS in PBS) were added to the plates, and the plates were incubated for 24 h at room temperature. After the plates were washed, biotin-labeled detection antibody and avidin-conjugated horseradish peroxidase (Av-HRP) were added to the plates, and the plates were incubated for 30 min at room temperature. Finally, the tetramethylbenzidine (TMB) substrate was added to the plates, and, after 30 min of incubation in the dark, TMB Stop Solution was added to stop the reaction. The optical density was measured at 450 nm on an automated ELISA reader. (Versa Max, Molecular Devices, CA, USA) [15].

### 2.8. Lung Wet to Dry (W/D) Weight Ratio

As the W/D ratio of the lung tissue is an important index for assessing the degree of pulmonary edema in ALI. We sought to examine the W/D ratio changes after different stimulations using a precision electronic scale (Denver, CO, USA). The lower lobe of the left lung was blotted dry and weighed before being placed in an oven at 80 °C for 48 h until a constant weight was obtained—as dry weight. The ratio of the wet lung weight to the dry lung weight was calculated to assess tissue edema.

### 2.9. Myeloperoxidase Activity

Lungs tissues were homogenized in sterile saline. The homogenate was centrifuged and the pellet was resuspended in a 50 mM K_2_HPO_4_ buffer (pH 6.0) containing 0.0005% hydrogen peroxide as a substrate, and 0.19 mg/mL of o-dianisidine chloride [16]. Spectral absorption is a measurement with a microplate reader at 460 nm. The myeloperoxidase (MPO) activity is expressed as optical density (OD) 460 nm/mg protein of lung tissue.

### 2.10. Western Blot Analysis

For western blot analysis, the cells or lung tissue (30–50 mg) was prepared into homogenate samples and lysed in a RIPA-lysis buffer with protease inhibitors, followed by centrifugation (12,000× *g*, 20 min) and the protein concentration determined by a Bio-Rad protein assay kit (BioRad, Hercules, CA, USA). Equal amounts of protein were subjected to 10% sodium dodecyl sulfate polyacrylamide gel electrophoresis (SDS-PAGE). Electrophoresed proteins were transferred onto PVDF membranes. After blocking, the membranes were incubated with primary antibody (1:2000 dilution). Appropriate horseradish peroxidase (HRP)-conjugated secondary antibodies (Sigma, St. Louis, MO, USA) were applied and the signals were detected using the enhanced chemiluminescent method (Amersham International plc., Buckinghamshire, UK). The western blot analysis was performed using Kodak Molecular Imaging Software (Eastman Kodak Company, Rochester, NY, USA). Strip and reprobe blots by stripping buffer (62.5 mM Tris-HCl (pH 6.7), 2% SDS, 100 mM β-mercaptoethanol) in a sealed bag at 50 °C for 30 min, followed by two washes in PBS-Tween for 10 min each. Membranes were then stripped and could be reprobed, beginning with the blocking step [17].

### 2.11. Statistical Analysis

Values are expressed as the mean ± standard error of the mean. One-way analysis of variance (ANOVA) or Student’s t-test was used to examine the differences among multiple groups or between two groups. ### denotes *p* < 0.001 compared with the control group; * denotes *p* < 0.05, ** denotes *p* < 0.01, and *** denotes *p* < 0.001 significant compared to the LPS-alone group.

## 3. Results

### 3.1. GRh2 Reduces LPS-Induced Histopathology Changes in Mice Lungs 

The morphology of the lungs was examined after LPS challenge. The results showed that the control group showed normal lung architecture. In the LPS-induced group, neutrophils infiltrated the pulmonary vessel, and edema of the interstitial space of the alveolar wall was observed causing alveolar epithelial cell damage. These pathological processes were improved by varying concentrations in GRh2 (5, 10, and 20 mg/mL) and Dex (10 mg/kg) in mice, suggesting that GRh2 alleviated the pathological effects in the LPS-induced ALI mouse model (Figure 1B,C). Furthermore, the lung-injury score showed GRh2 improved LPS-induced inflammatory response. These results suggest that GRh2, evidenced by reduced inflammatory cell infiltration, protected the LPS challenge histopathological changes in the lung tissues of mice.

### 3.2. Decreased Pulmonary Wet/Dry Weight Ratio and MPO Activity 

**Figure 2:**
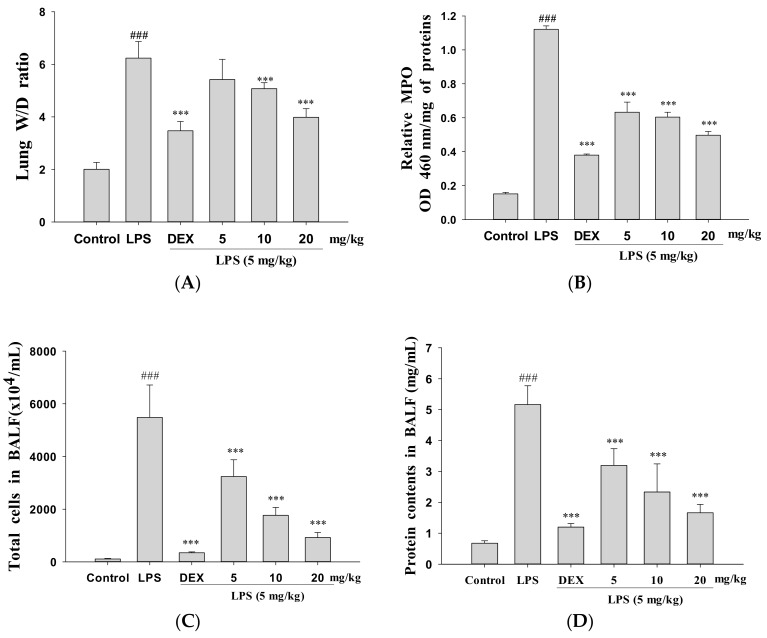
GRh2 improved (**A**) pulmonary edema (wet-dry (W/D) ratio); (**B**) myeloperoxidase (MPO) activity in vivo; and reduced (**C**) cellular counts and (**D**) total protein in bronchoalveolar lavage fluid (BALF). Lung tissues were weighed and calculated the W/D ratio. BALF was harvested to investigate at 6 h after LPS treatment. Total cells and total proteins in BALF were analyzed. Data are presented as mean ± S.E.M. (*n* = 5). ### denotes *p* < 0.001 compared with sample of control group. *** denotes *p* < 0.001 compared with the LPS-only group.

The effect of GRh2 was determined using the pulmonary W/D ratio and MPO activity on LPS-induced lung injury in mice. Under LPS stimulation, the lung vascular permeability caused by edema compared improved to the control group as indicated in the lung W/D ratio. However, GRh2 and Dex treatment reduced the lung W/D ratio compared with the group treated with LPS alone (Figure 2A). These results showed that GRh2 could eliminate lung edema and pulmonary inflammation after LPS challenge.

Neutrophil infiltration can increase inflammatory responses and cell injury. However, MPO activity is a more useful index of neutrophil influx into lung tissue [18]. As Figure 2B illustrates, mice were exposed to a significant increase in MPO activity via intratracheal instillation of LPS in lung tissues. When pretreated with GRh2 (5, 10, and 20 mg/mL) and Dex, MPO activity decreased in the LPS-only treatment mice. These data revealed that GRh2 prevented pulmonary edema and lung tissue infiltration by LPS-challenged mice. 

### 3.3. Decreased Total Cell Count and Protein Concentration 

LPS caused a marked increase in total cells compared to the control group. However, pretreatment with GRh2 or Dex significantly decreased in the total cell count compared with LPS-induced ALI mice (Figure 2C). Compared with data from the control group, total protein concentration in BALF decreased significantly after pretreatment with GRh2 and Dex (Figure 2D). These results demonstrated that the inhibitory effect of GRh2 on ALI is connected with the decrease in leukocytes sequestration and the inflammatory response in the lung tissues.

### 3.4. Decreased Proinflammatory Cytokine Levels 

**Figure 3:**
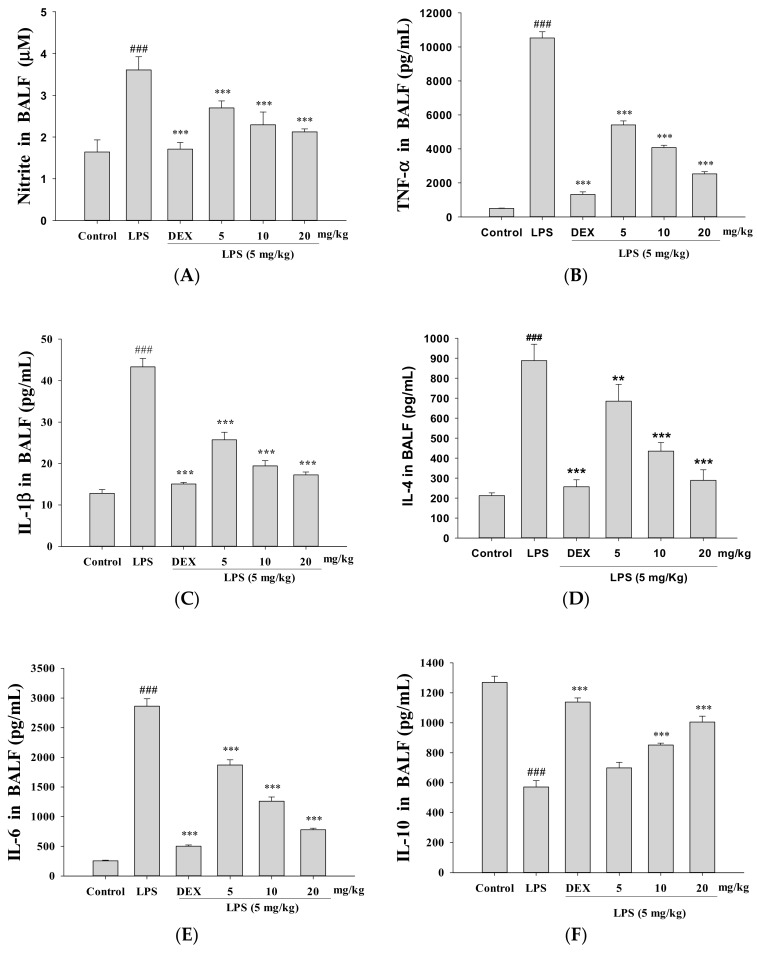
GRh2 downregulated (**A**) nitric oxide (NO); (**B**) tumor necrosis factor-alpha (TNF-α); (**C**) IL-1β; (**D**) IL-4; and (**E**) IL-6; and (**F**) increased IL-10 in BALF. BALF was collected. NO, TNF-α, IL-1β, IL-4, IL-6, and IL-10 were detected at 6 h after LPS challenge by enzyme-linked immunosorbent assay (ELISA). Data are represented as mean ± S.E.M. (*n* = 5). ### denotes *p* < 0.001 compared with sample of control group. ** *p* < 0.01 and *** *p* < 0.001 compared with LPS-only group.

Inflammatory mediator levels in tissues were detected by ELISA. LPS-stimulated ALI mice significantly increased the NO, TNF-α, IL-1β, IL-4, and IL-6 levels in BALF compared to the control group (Figure 3A–E, respectively). GRh2 and Dex treatment improved NO, TNF-α, IL-1β, IL-4, and IL-6 production after LPS challenge. Furthermore, the GRh2-treated group in LPS-induced ALI BALF significantly increased IL-10 concentration (Figure 3F).

### 3.5. Inhibition of LPS-Induced ALI iNOS and COX-2 and Inactivation of NF-κB and IκBα Protein Expressions

We examined whether pretreatment with GRh2 would inhibit NO production and iNOS and COX-2 protein expressions after an LPS challenge. The results revealed that pretreatment with GRh2 inhibited the protein expression of iNOS and COX-2 after LPS challenge of lung tissues.

**Figure 4:**
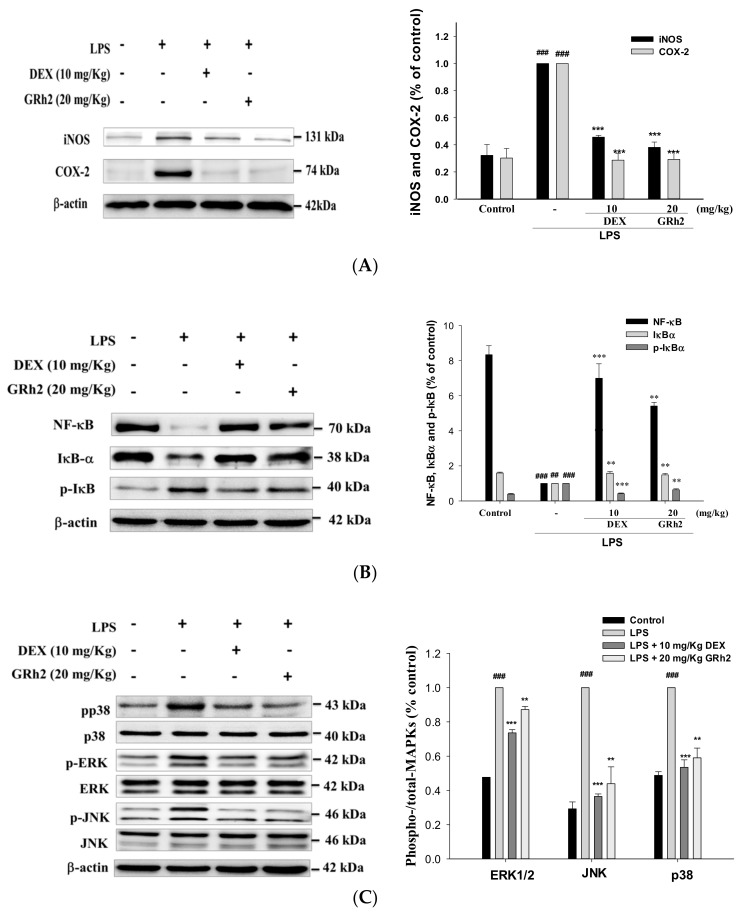
Effects of GRh2 on LPS-induced (**A**) iNOs, COX-2; (**B**) IκB-α, NF-κB; and (**C**) mitogen-activated protein kinase (MAPK) phosphorylation signaling expression in lungs. Protein levels of iNOs, COX-2, IκB-α, NF-κB, and MAPK phosphorylation protein expression in lung homogenates were evaluated by western blot analysis after LPS challenge 6 h later. Densitometric analysis of the relevant bands was performed. Data are represented as mean ± S.E.M. (*n* = 2). ## *p* < 0.01 and ### *p* < 0.001 compared with the control group. ** *p* < 0.01 and *** *p* < 0.001 compared with LPS-only group.

The signal pathways caused the NF-κB accumulation in the nucleus, which can be activated by a variety of stimuli such as TNFα and IL-1β [6]. Our study demonstrates that GRh2 treatment inhibited the IκBα and NF-κB degradation in LPS-challenged mice (Figure 4A,B). Thus, GRh2 regulates the NF-κB signaling pathway after LPS challenge. 

### 3.6. Suppression of the MAPK Pathway Activation 

As shown in Figure 4C, we found that the phosphorylation of MAPK proteins was activated after LPS challenge in mice. Moreover, the expression of phosphorylated ERK, JNK, and p38 decreased when pretreated with GRh2 and Dex. These results revealed that GRh2 suppresses the expression of MAPK proteins after LPS challenge. 

### 3.7. Decreased LPS-Induced Oxidative Stress and HO-1/Trx-1/KAP-1/Nrf2 Signaling Pathway 

**Figure 5:**
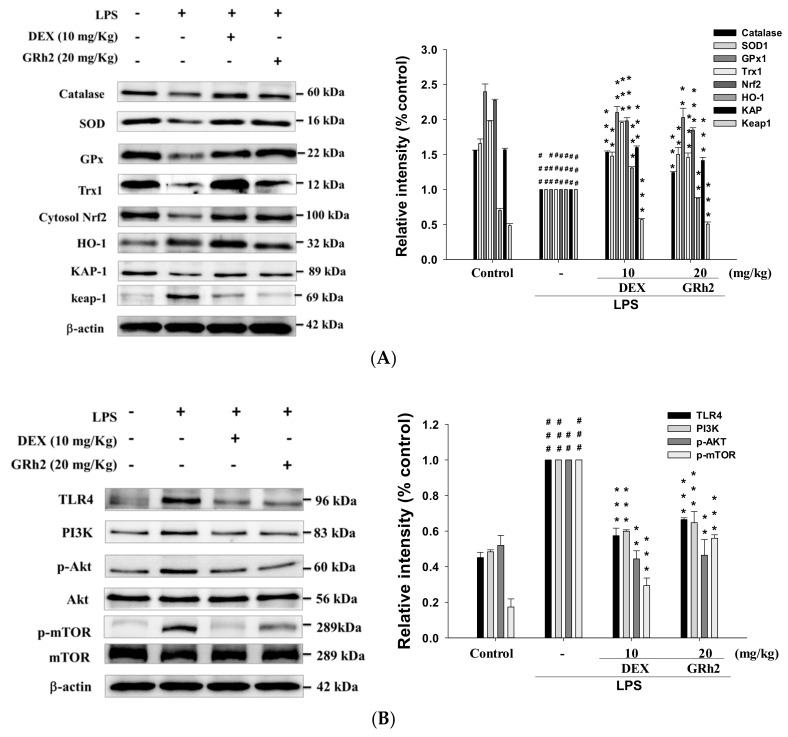

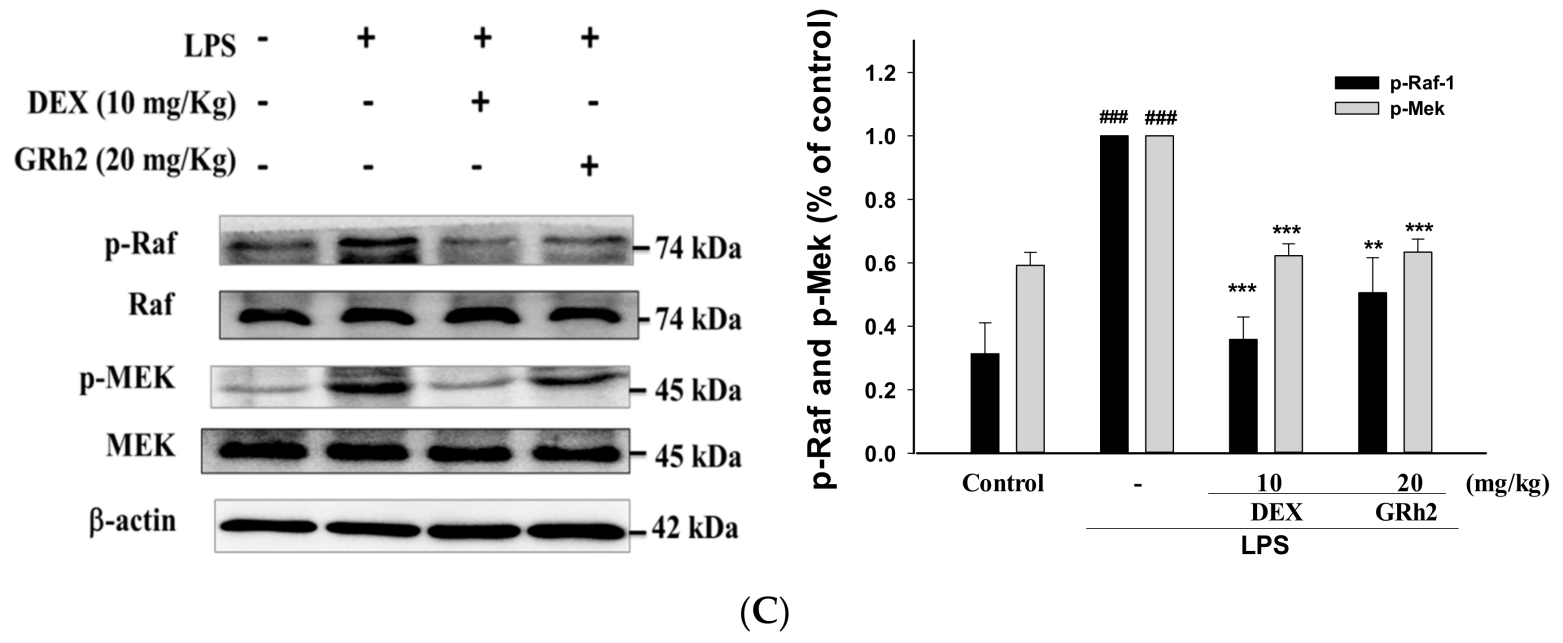
Effects of GRh2 on (**A**) LPS-induced antioxidative enzymes (catalase, superoxide dismutase (SOD), and glutathione peroxidase (GPx)); heme oxygenase-1 (HO-1), Trx-1, NF-E2-related factor 2 (Nrf2)/Keap1 and KAP1; (**B**) TLR4, PI3K, Akt, and mTOR; and (**C**) Raf-1, p-Raf-1, Mek and p-Mek protein expression in the lungs. Protein levels of catalase, SOD, GPx, HO-1, Trx-1, Nrf2/Keap1, KAP1, TLR4, PI3K, Akt, mTOR, Raf-1, p-Raf-1, Mek and p-Mek protein expression in lung homogenates were evaluated by western blot analysis after LPS challenge 6 h hours later. Densitometric analysis of the relevant bands was performed. Data are represented as mean ± S.E.M. (*n* = 2). ## *p* < 0.05 and ### *p* < 0.001 compared with sample of control group. ** *p* < 0.01 and *** *p* < 0.001 compared with LPS-only group.

Oxidative stress elevated the levels of reactive oxygen species that cause tissue injury. Superoxide dismutase (SOD) is a class of related enzymes that catalyze the breakdown of superoxide anions, and SOD activity was reduced in LPS-induced ALI mice [7]. LPS administration alone reduced the activity of catalase, SOD, and glutathione peroxidase (GPx) (Figure 5A). The antioxidative relative protein expressions of HO-1, Trx-1, KAP-1, and Nrf2, and elevated Keap1 protein expression, compared with the control group (Figure 5A). However, GRh2 increased the antioxidant enzyme activities and the antioxidative relative protein expressions compared with the LPS-only group. These results demonstrated that GRh2 improved the expression of antioxidative-enzyme-related proteins after LPS challenge. 

### 3.8. Decreased TLR4/PI3K/Akt/mTOR Signaling 

As shown in Figure 5B, the LPS-only group demonstrated an increased expression of TLR4, PI3K, Akt, and mTOR proteins compared with control groups. GRh2 treatment inhibited the expression of the TLR4/PI3K/Akt/mTOR signal transduction pathway compared with the LPS-induced group. These results highlight the protective effect of GRh2 after LPS challenge through the inhibition of the protein expression of the TLR4/PI3K/Akt/mTOR signal pathway. 

### 3.9. Activation of the RAF/MEK Pathway 

Growth factors use the Raf/MEK signaling cascade to transmit signals to regulate protein expression. In this study, LPS-only treatment increased the phosphorylation of RAF and MEK protein expression compared with the control group (Figure 5C). On the contrary, GRh2 administration significantly inhibited the activity of the RAF/MEK pathway when compared with the group treated with LPS alone. These results suggest that GRh2 inhibited the RAF/MEK pathway after LPS challenge. 

### 3.10. Blocking RAF Synergy with GW-5074 to Increase Anti-Inflammatory Capacity of GRh2 

**Figure 6:**
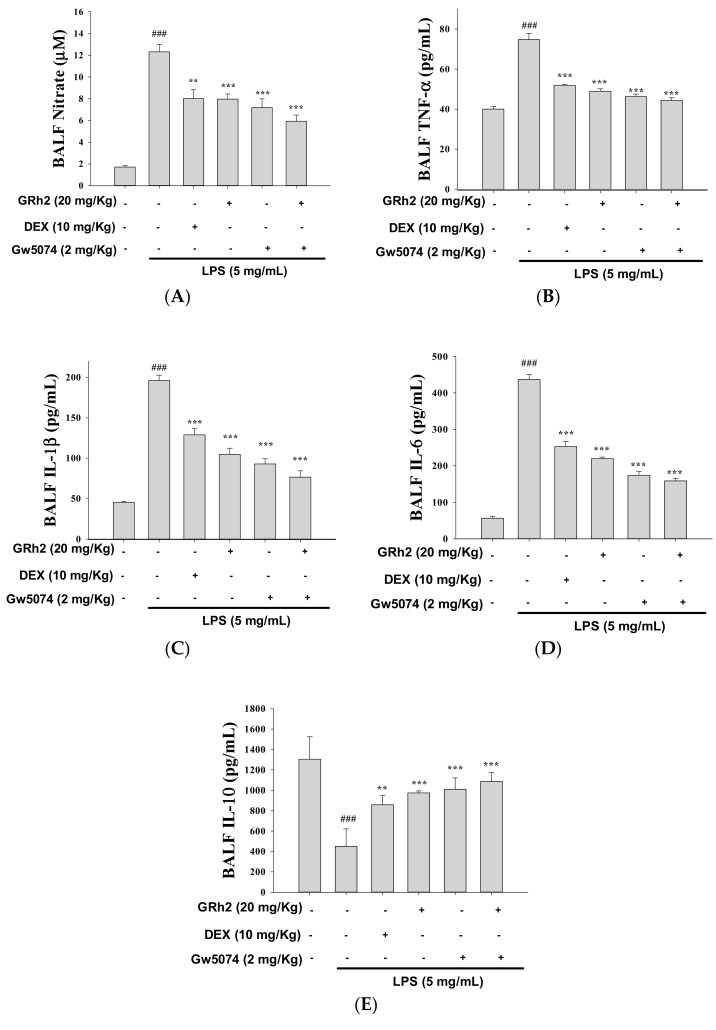
(**A**) GRh2 and Raf-1 inhibitor (GW-5074) reduced NO; (**B**) TNF-α; (**C**) IL-1β; (**D**) IL-6; and (**E**) IL-10 in BALF. BALF was collected. NO, TNF-α, IL-1β, IL-6, and IL-10 were detected at 6 h after LPS challenge by ELISA. Data are represented as mean ± S.E.M. (*n* = 5). ### *p* < 0.001 compared with sample of control group. ** *p* < 0.01 and *** *p* < 0.001 compared with LPS-only group.

To determine whether the Raf-1 kinase inhibitor (GW-5074) could suppress the Raf-1/MEK/ERK pathway, we investigated the patterns of the protein expression related to the Raf-1/MEK/ERK pathway in LPS-challenged mice treated with GW-5074 (2.0 mg/kg). The LPS-induced proinflammatory-cytokines release (NO, TNF-α, IL-1β, and IL-6) was suppressed by GW-5074 inhibitor. In addition, the induction of proinflammatory cytokines was inhibited by cotreatment with GRh2 and GW-5074 compared to the LPS-alone group (Figure 6A–D). Furthermore, cotreatment of GRh2 with GW-5074 significantly increased the IL-10 concentration in BALF (Figure 6E). These results indicate that GRh2 suppressed the activity of Raf-1/MEK/ERK pathways in LPS-challenged ALI mice. 

## 4. Discussion

ALI is a severe form of diffuse lung disease described as a clinical syndrome of acute respiratory failure with high morbidity and mortality [9]. It is characterized with persistent pulmonary inflammation [10] and increase in microvascular permeability [11]. Even with patients surviving ALI, the quality of life remains poor. Therefore, there is a great need for more effective therapeutic approaches. In this study, we induced ALI in an animal model using intratracheal instillation of LPS challenge to confirm the anti-inflammatory protective effect of GRh2. LPS caused significant and dose-related increases in proinflammatory-cytokines production, which occurred after two to six hours, to a maximum of 24 h [19]. Glucocorticoids are a class of steroid hormones that may be used as anti-inflammatory medicines in the treatment of most patients with ALI. Therefore, the positive control of Dex highlighted the efficacy of GRh2 as an anti-inflammatory agent in LPS-challenged ALI mice. Nonsteroidal anti-inflammatory drugs are among the most commonly used drug classes to reduce pain and inflammation. The discovery and development of anti-inflammatory drugs must be based on their effects on signal transduction and as anticytokine agents [20,21]. Inflammation is a key factor in the pathogenesis of ALI. Therefore, suppression of inflammatory response could be a potential strategy to treat LPS-induced lung injury. GRh2 has been reported to attenuate allergic airway inflammation through an anti-inflammatory mechanism [18], but its effect on ALI is poorly understood. Thus, we found that GRh2 treatment reduced LPS-induced lung edema, neutrophil infiltration, secretion of proinflammatory cytokines in the BALF, and regulation of the Raf-1/MEK/ERK and Keap1/Nrf2/HO-1 signaling pathways. This study indicates that GRh2 exhibits anti-inflammatory and antioxidant activity after LPS challenge.

ALI is characterized by a disruption in the integrity and function of the endothelial and epithelial barriers, pulmonary edema, release of proinflammatory cytokines and inflammatory mediators, and large numbers of neutrophils infiltration [22]. LPS-challenged ALI mice have been extensively used to study pathological processes and states [23]. Thus, the ALI animal model induced by administration of LPS through the trachea is suitable for the study of potential human primary prevention or treatment drugs.

LPS-induced ALI induced a large number of proinflammatory mediators to recruit neutrophils to the lungs [24]. In the early stages of the inflammatory response, neutrophils are the main class of immune cells that destroy the alveolar capillaries. Ultimately, neutrophils-infiltration dysfunction leads to hypoxia and pulmonary edema associated with the formation of a hyaline membrane in alveolar walls [25]. The clinical pathological features of ALI in humans are similar to LPS challenge ALI in the murine model [26]. However, the search for an effective pharmacologic therapy for ALI is ongoing. Therefore, we used intratracheal instillation to study the role of GRh2 in the LPS challenge ALI mouse model and to establish a new drug for ALI. We found that GRh2 mitigated the LPS-induced histopathological changes, including infiltration of proinflammatory cells and lung edema.

The main pathogenesis of LPS-induced ALI is via the infiltration of leukocyte and macrophage migration into lung tissues [27]. In the current study, GRh2 treatment markedly reduced the total cell numbers compared to LPS-treated animals. In addition, MPO is the most abundant granule enzyme in the neutrophil and plays a central role in inflammatory disorders. The measurement of MPO activity is a quantitative assessment for evaluating neutrophils infiltration into lung parenchyma or alveolar spaces [28].

Cytokines are a superfamily of related low-molecular-weight proteins that mediate many of the immune reactions where the cytokine signals are amplified [29]. Proinflammatory cytokines are released by activated macrophages to play a critical early role in inflammatory diseases as demonstrated in several clinical studies. ALI is caused by many proinflammatory cytokines, including TNF-α, IL-1β, IL-4, IL-6, and IL-10 and other anti-inflammatory cytokine mediators [30]. Increased levels of proinflammatory cytokines have been observed in ALI patients and are associated with major inflammatory disorders [31]. TNF-α is a key cytokine exerting pleiotropic effects and can induce a proinflammatory response via high particle concentrations. In addition, IL1 and its related family members are mainly proinflammatory cytokines associated with the pathogenesis of acute and chronic disorders [32]. In addition, IL-1β stimulates the production of other cytokines in the inflammatory response and modulates the proinflammatory cytokine cascade in ALI [33]. IL-6 is released by monocytes and macrophages in response to other cytokines in diseases involving inflammatory response. In a clinical test, higher levels of IL-6 were observed in the serum of ALI patients that predicted increased mortality [34]. After LPS induction, an increase in IL-4 content in BALF at 24 h and IL-4 expression was found to be correlated with the deposition of extracellular matrix and lung fibrosis [35]. These results demonstrate that remodeling processes often cause unregulated fibroproliferation and fibrosis in late-phase ALI [28]. In addition, the major function of IL-10 is to strongly inhibit the production of proinflammatory cytokines. Studies have suggested that IL-10 protects against lethality during endotoxemia-induced shock in mice [35]. IL-10 promoters differ in the absence of an NF-κB binding site and the presence of a cyclic AMP response element [35]. In our study, the levels of TNF-α, IL-1β, IL-4, and IL-6 were dramatically decreased and IL-10 levels were elevated by GRh2 in BALF. 

Cells contain a number of antioxidant enzymes (SOD, catalase, and GPx) to prevent cell or tissue damage caused by the expression of antioxidant enzymes in an attempt to decrease oxidative stress. SOD is an efficient enzyme that catalyzes the partitioning of the superoxide anion to hydrogen peroxide and oxygen, whereas catalase and GPx catalyze hydrogen peroxide to form oxygen and water [10]. In this study, we showed that GRh2 increases the antioxidant protein activity (SOD, catalase, and GPx) in the ALI model. In addition, studies showed that Keap1 is a key sensor for oxidative stress. Under oxidative stress, Nrf2 is released from Keap1, which activates Nrf2 and its downstream-regulated genes in the nucleus. Nrf2 is correlated with the induction of HO-1, GPx, glutathione-S-transferase, and Trx-1, allowing the scavenging of free radicals in cells caused by oxidative damage [18]. Nrf2 functions to maintain cellular homeostasis through its ability to regulate antioxidant proteins, detoxification enzymes, and other stress-response proteins [18]. In addition, PI3K and MAPK signaling are connected in the activation of Nrf2 [36]. The results indicated that the protective effects of GRh2 regulated the Keap1/Nrf2/HO-1 signal pathway against oxidative stress. Nrf2 is a key molecule involved in targeting specific proteins in the NF-κB pathway that may be associated with inflammatory regulation.

NF-κB is a family of transcription factors that serves as signal regulators in inflammation, and cell proliferation and differentiation [18]. Activation of NF-κB was reported to increase with ALI in a clinical test, and NF-κB p65 overexpression was found in the alveolar macrophages in ALI patients, caused by severe infection compared to the control group [7]. In addition, MAPK cascades have been proven to play a vital role in the transduction of extracellular signals to cellular responses such as inflammatory-cytokine production induced by LPS. 

MAPK was activated immediately after LPS, and the levels of phosphorylated MAPKs (ERK1/2, p38, and JNK) increased [12]. In this study, GRh2 suppressed the phosphorylation of MAPKs in LPS-challenged ALI mice. GRh2 also suppressed the TNF-α, IL-1β, and IL-6 production through NF-κB activation because they control proinflammatory-cytokine expression in the LPS-induced model. In this research, we discovered that GRh2 significantly inhibited IκB-α degradation and the phosphorylation of NF-κB and MAPK in LPS-challenged mice. 

PI3K/AKT/mTOR signaling plays an active role in regulating several of cellular functions, including cell proliferation and apoptosis, and is critically modulated in TLR signaling pathways [36]. This result showed that the inflammatory response in LPS-induced ALI mice was mediated through the TLR4 receptor, which increased the levels of PI3K, p-Akt, and p-mTOR protein expression. The results of our study confirm that the PI3K/AKT/mTOR pathway is a potential predictive marker for GRh2 treatment by LPS-challenged mice.

The activation of the ERK pathway is caused by various upstream stimuli that accumulate on the Ras family of small G proteins [19]. Activated Ras then engages with its downstream effectors. A series of kinases from RAF to MEK to MAPK is an example of a protein kinase cascade. Raf-1 is a serine threonine kinase that phosphorylates and activates a family of protein kinases termed MAP kinase or Mek [37]. The Raf-1-mediated inflammatory signaling plays a key role in the inflammatory response. The specific inhibitor of Raf-1, GW5074, reduces the activation of these target pathways, and has been shown to completely inhibit proliferation and apoptosis in several cells types [38]. In the present study, the effects of LPS-induced proinflammatory-cytokine release were suppressed by GRh2 and Raf-1 inhibitor. The Raf-1 inhibitor, GW5074, significantly suppressed the inflammatory responses. This agrees well with other reports that GW5074 reduces airway inflammation [39]. The Ras/Raf-1/MAP pathway is thought to play an active role in the modulation of cell survival, proliferation, and differentiation.

In this study, our results clearly show that TLR4 mediates the LPS-induced activation of ERK1/2 proteins via Ras/Raf-1/MAP kinase. The activation of Raf-1 is quick and leads to the activation of Mek. Moreover, based on our studies of the Raf-1 kinase inhibitor, LPS participates in the TLR4 induction of the canonical Raf/Mek/ERK pathway. Multiple points of contact exist between PI3K/Akt/mTOR and Raf/Mek/ERK signaling. For example, the activation of NF-κB is associated with the TNFα receptor and the Ras/Mek/ERK pathways couple to regulate the NF-κB-dependent production of pro-inflammatory mediators [40,41]. Therefore, these results demonstrate that Raf/Mek/ERK signaling pathways were stimulated in this LPS-challenge ALI animal model, and this pathway can be blocked with GRh2 treatment.

## 5. Conclusions

**Figure 7:**
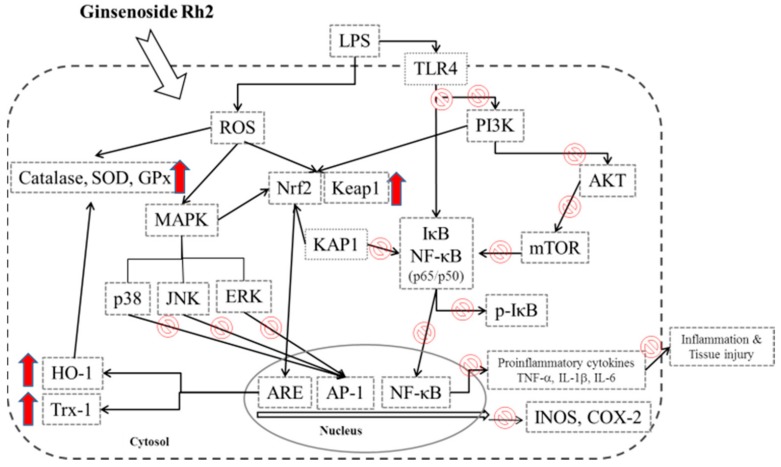
The schemes of the mechanism for the protective effect of GRh2 on LPS-induced inflammation.

In conclusion, our study showed that GRh2 regulates inflammatory responses in an LPS-challenge animal model by inhibiting lung pathologic changes, lung edema, inflammatory cell infiltration, and the release of a variety of proinflammatory cytokines (TNF-α, IL-1β, IL-4, and IL-6), and increased the release of IL-10. Together, the research data suggested that GRh2 has potent anti-inflammatory properties by inhibiting the TLR4/ PI3K/Akt/mTOR, Raf-1/MEK/ERK, and Keap1/Nrf2/HO-1 signaling pathways (Figure 7). Therefore, GRh2 exerts anti-inflammatory effects in vivo and requires more comprehensive research before realizing the full clinical application of the drug.
